# d-Xylitol Production from Sugar Beet Press Pulp Hydrolysate with Engineered *Aspergillus niger*

**DOI:** 10.3390/microorganisms12122489

**Published:** 2024-12-03

**Authors:** Melanie Knesebeck, Marcel Rüllke, Veronika Schönrock, J. Philipp Benz, Dirk Weuster-Botz

**Affiliations:** 1 Biochemical Engineering, TUM School of Engineering and Design, Technical University of Munich, Boltzmannstraße 15, 85748 Garching, Germany; 2 Fungal Biotechnology in Wood Science, TUM School of Life Sciences, Technical University of Munich, Hans-Carl-von-Carlowitz-Platz 2, 85354 Freising, Germanybenz@hfm.tum.de (J.P.B.)

**Keywords:** d-xylitol production, *Aspergillus niger* NRRL3, sugar beet press pulp hydrolysate, l-arabinose, bioconversion

## Abstract

d-Xylitol is a low-calorie and anti-cariogenic sweetener suitable for diabetic patients, making it a valuable ingredient in various health-related applications. In this study, we investigated the production of d-xylitol from l-arabinose derived from sugar beet press pulp (SBPP) hydrolysate using an engineered *Aspergillus niger* strain. Initial batch studies applying stirred tank bioreactors demonstrated d-xylitol production of 4.6 g L^−1^ with a yield of 0.37 g d-xylitol g^−1^ l-arabinose with a synthetic medium. Subsequently, the conversion of enzymatically produced and clarified SBPP hydrolysate was studied. We found that pre-treatment of the enzymatic hydrolysate with activated carbon was essential to remove inhibitory components. Moreover, an automated aeration switch-off was implemented based on the CO_2_ signal of the off-gas analyzer of the stirred tank bioreactor to prevent d-xylitol degradation after l-arabinose depletion. This resulted in a final d-xylitol concentration of 4.3 g L^−1^ with an improved yield of 0.43 g d-xylitol g^−1^ l-arabinose. The feasibility of utilizing the agricultural residue SBPP for d-xylitol production was successfully demonstrated with engineered *A. niger*.

## 1. Introduction

The increasing world population needs more food despite finite resources, e.g., arable land. Concurrently, the pressing issue of climate change mandates a worldwide effort to reduce carbon dioxide emissions from fossil resources [1]. The circular economy has a high potential to reduce CO_2_ emissions by using renewable sources to produce commodities [2]. One crucial aspect is the competition of land use for food production or providing renewable plant materials for other applications. Using agricultural residues avoids this competition, and the overall quantities tend to be large, although the sources and their composition are very region-specific [3,4,5]. 

One promising agricultural residue is extracted sugar beet press pulp (SBPP). In 2019, about 14 million tons of SBPP (dry matter) remained after sucrose extraction [6]. SBPP consists of different sugar polymers, such as cellulose, hemicellulose, and pectin. The main monomeric compounds are d-glucose, d-galacturonic acid, and l-arabinose, among other sugars [7,8]. For further valorization of the raw material, chemical and/or enzymatic hydrolysis is necessary to make use of the monomeric sugar compounds. 

In a previous study, enzymatic hydrolysis of SBPP led to a total sugar concentration of nearly 55 g L^−1^ with enzymes produced by an engineered *Aspergillus niger* strain in a one-pot process combining solid-state fermentation for enzyme production followed by hydrolysis of SBPP in the same stirred tank bioreactor [9]. The released sugars can be used for further biotransformation. d-Galacturonic acid can be used for l-galactonate production [10,11], which was shown to have promise as an alternative leavening agent in baking applications [12]. Another key component of SBPP is l-arabinose, which achieved an 86% yield (final concentration of 18.8 g L^−1^) after enzymatic hydrolysis in the previously mentioned one-pot process [9].

d-Xylitol is a highly promising product with diverse health applications. Its anti-cariogenic properties and low-calorie content make it suitable for various uses, including chewing gum and toothpaste, and as a dietary option for individuals with diabetes and obesity [13]. Additionally, d-xylitol has the potential to serve as a platform chemical to produce various common products traditionally derived from fossil sources, such as propylene glycol, glycerol, xylaric acid, and others. Market projections anticipate the value of d-xylitol to reach USD 6.93 billion by 2027 [14].

To date, d-xylitol is primarily derived from birch wood through a chemical production process involving four main steps. This includes extracting d-xylose from birch wood biomass through acid hydrolysis, purifying the hydrolysate to isolate d-xylose, and catalytically hydrogenating d-xylose to d-xylitol, which is finally isolated by crystallization. However, this process is known for its high energy consumption, resulting in high production costs. An alternative biotechnological approach involves genetically modified microorganisms, such as yeast, to convert d-xylose to d-xylitol, which presents a promising environmentally friendly approach [14].

A recent study introduced another biotechnological approach for producing d-xylitol using l-arabinose instead of d-xylose. A genetically modified *A. niger* strain with specific enzyme knockouts (d-xylitol-dehydrogenase, d-sorbitol dehydrogenase, and d-xylulose kinase) to prevent degradation of d-xylitol and the constitutive heterologous expression of an aquaglyceroporin gene shown to facilitate the export of d-xylitol enabled the production of d-xylitol from l-arabinose with a synthetic medium in shake flasks with a yield of 0.45 g d-xylitol g^−1^ l-arabinose [15,16,17]. The reduced d-xylitol yield indicates that the engineered *A. niger* strain can still metabolize d-xylitol [17].

In the present study, this engineered *A. niger* strain was tested for d-xylitol batch production from l-arabinose with clarified SBPP hydrolysate [9] in a scalable stirred tank bioreactor. The l-arabinose in the SBPP hydrolysate is converted to d-xylitol, whereas the other sugars in the hydrolysate serve as a carbon source for the growth of *A. niger* in the batch process. Applying such a simple whole-cell biotransformation process makes it necessary to prevent the re-consumption of d-xylitol. Since this is an aerobic process, we studied a control strategy to stop this re-consumption by switching off the aeration automatically based on the CO_2_ concentration in the exhaust gas. In addition, we evaluated the usability of clarified SBPP hydrolysate without, and if necessary, with pre-treatment to ensure efficient conversion of l-arabinose in the SBPP hydrolysate.

## 2. Materials and Methods

### 2.1. Fungal Strain

The genetic engineering of the *Aspergillus niger* NRRL3 strain was described before (triple knock-out of the genes Δ*xdhA*, Δ*sdhA*, Δ*xkiA*, and the integration of a constitutively opened Fps1 transporter from *Saccharomyces cerevisiae* combined with the promoter P*tvdA*) [17]. The engineered strain *A. niger* ΔΔΔ P*tvdA*-*FPS1_open_* was shown to convert l-arabinose to d-xylitol with a synthetic medium on a shake flask scale. The Fps1 transporter was necessary for the d-xylitol export into the medium.

### 2.2. Sugar Beet Press Pulp Hydrolysate

The extracted sugar beet press pulp (SBPP) hydrolysate was produced in a one-pot process with dried, non-milled (particle size 0.5 mm < x < 6.3 mm), and milled SBPP (particle size x < 2 mm) provided by Südzucker AG (Mannheim, Germany) [9]. The produced hydrolysate was centrifuged for 30 min at 14,000 RCF (Rotana 460 R, Andreas Hettich GmbH, Tuttlingen, Germany) and 4 °C. The supernatant was filtered at 11 µm and subsequently at 2.7 µm (Whatman^TM^ filter papers grade 1 and grade 5, GE Healthcare GmbH, Solingen, Germany), and stored at −20 °C until usage in sterile Whirl-pak^®^ sample bags (neoLab Migge GmbH, Heidelberg, Germany).

The SBPP hydrolysate was used for cultivation experiments either directly after filtration, after heat sterilization at 121 °C for 20 min, or after pre-treatment with activated charcoal (X865.2, Carl Roth GmbH, Karlsruhe, Germany). The activated charcoal extraction was performed with 1584 mL SBPP hydrolysate and 140 g activated charcoal. Afterward, the suspension was centrifuged for 20 min at 4 °C and 14,000 RCF (Rotana 460 R, Andreas Hettich GmbH, Tuttlingen, Germany). The supernatant was filtered with 11 µm first and 2.7 µm pore size second (Whatman^TM^ filter papers grade 1 and grade 5, GE Healthcare GmbH, Solingen, Germany) to remove the residue charcoal. Finally, the SBPP hydrolysate was sterile-filtered with a 0.22 µm Steritop^®^ bottle-top filter (Merck KGaA, Darmstadt, Germany).

### 2.3. Preculture, Inoculation, and Cultivation Medium

The preculture of the engineered *A. niger* strain was carried out by adding 40 µL of glycerol stocks to 39 g L^−1^ potato extract glucose agar plates (Carl Roth GmbH+Co.KG, Karlsruhe, Germany) supplemented with 10 g L^−1^ yeast extract and final concentrations of 1 mM uridine, and 1 mL L^−1^ of trace elements solution. The trace elements solution (L^−1^) consisted of the following: 10 g EDTA, 4.4 g ZnSO_4_ · 7 H_2_O, 1.01 g MnCl_2_ · 4 H_2_O, 0.32 g CoCl_2_ · 6 H_2_O, 0.32 g CuSO_4_ · 5 H_2_O, 0.22 g (NH_4_)_6_Mo_7_O_24_ · 4 H_2_O, 1.47 g CaCl_2_ · 2 H_2_O, and 1 g FeSO_4_ · 7 H_2_O [18]. The *A. niger* spores were harvested after 5 days of incubation at 30 °C by adding sterile 0.89% (*w/v*) NaCl solution with 0.05% (*v/v*) Tween 80. Afterward, the spore solution was filtered with sterile cotton wool before diluting with NaCl-Tween 80 solution to achieve a defined spore concentration.

The cultivations with synthetic medium were performed with 12 g L^−1^ d-glucose and 13 g L^−1^ l-arabinose. Additionally, the following components necessary for the growth were added to the medium with final concentrations of 175 mM (NH_4_)_2_SO_4_, 7 mM KCl, 11 mM KH_2_PO_4_, 2 mM MgSO_4_, 1 mM uridine, 1 mL L^−1^ polypropylene glycol P2000, and 1 mL L^−1^ trace element solution. The cultivations with SBPP hydrolysate contained 600 mL hydrolysate and 93 mL of the previous components necessary for growth with the same final concentrations. The inoculation volume with spore solution was 7 mL.

The pre-culture in shake flasks was carried out with synthetic medium inoculated with 10^9^ spores L^−1^ and incubated for 96 h at 30 °C and 250 min^−1^ (WIS-20R Incubator, Witeg Labortechnik GmbH, Wertheim, Germany). The stirred tank bioreactors were inoculated each with 10% (*v/v*) pre-culture of the final volume.

### 2.4. Cultivation in Stirred Tank Bioreactors

A fourfold parallel stirred tank bioreactor unit (DASGIP^®^, Eppendorf SE, Hamburg, Germany) was used with 0.7 L medium each for the batch cultivations of the d-xylitol-producing *A. niger* strains in duplicates. Each of the baffled stirred tank bioreactors with an inner diameter of 11 cm and a liquid height of 20 cm was equipped with one Rushton turbine at a height of 8 cm, and one elephant’s ear stirrer 3 cm above the vessel bottom (both stirrers DASGIP^®^, Eppendorf SE, Hamburg, Germany). The bioreactors were inoculated with 10^9^ spores L^−1^ each. The aeration started after 3 h, and the initial stirrer speed of 250 min^−1^ prevented spore loss. The following reaction conditions were kept constant at 30 °C, an aeration rate of 0.2 volume air per volume medium per minute (vvm) with sterile air, and an agitation rate of 600 rpm. A constant pH of 4.5 was controlled by adding 1 M KOH or 0.5 M H_2_SO_4_. The exhaust gas’s carbon dioxide and oxygen composition were monitored online (BlueVary, BlueSens Gas Sensor GmbH, Herten, Germany). The aeration automatically stopped when the CO_2_ concentration in the exhaust gas dropped below 0.6% in the processes with synthetic medium and 0.9% with SBPP medium.

### 2.5. Quantification of Sugars and Organic Acids in Cultivation Supernatants

The detailed method for quantifying the sugars and organic acids from cultivation samples was already described in a previous study [9]. Shortly, the concentrations were measured by HPLC (1100 Series, Agilent Technologies, Santa Clara, CA, USA) with a cation exchange column (Rezex ROA-Organic Acid, Phenomenex Ltd., Aschaffenburg, Germany) and a constant flow rate of 0.5 mL L^−1^ of the eluent 5 mM H_2_SO_4_.

### 2.6. Gravimetric Determination of the Biomass Dry Weight

The biomass dry weight concentration was determined in triplicates with 1 mL of cultivation supernatant, each in pre-weighted and dried sampling tubes (Safe-Lock Tube 1.5 mL, Eppendorf SE, Hamburg, Germany). These tubes were centrifuged for 10 min at 20,000 RCF at 4 °C (Centrifuge 5424 R, Eppendorf SE, Hamburg, Germany), and the supernatant was discarded. The remaining biomass was dried at 80 °C for at least 48 h. After cooling down, the tubes were weighed (analytical balance XA204, Mettler-Toledo GmbH, Gießen, Germany) again, and the biomass dry weight was calculated.

## 3. Results and Discussion

### 3.1. d-Xylitol Production with Synthetic Medium

**Figure 1:**
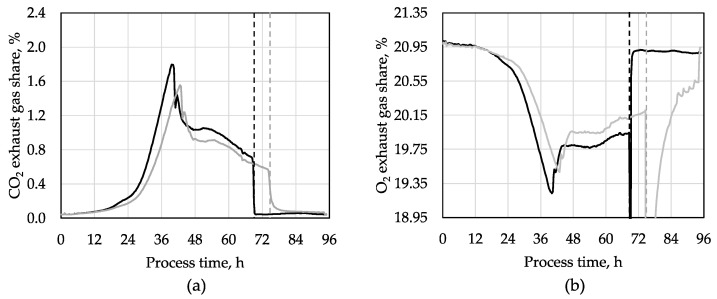
d-xylitol production by engineered *A. niger* in stirred tank bioreactors with synthetic medium: (**a**) carbon dioxide (CO_2_), and (**b**) oxygen (O_2_) concentrations in the exhaust gases of two parallel batch processes with the d-xylitol-producing *A. niger* strain (pH 4.5, 30 °C, 0.2 vvm aeration after 3 h until the total consumption of l-arabinose was detected online by the rapid decrease in CO_2_ production, indicated by the dashed lines).

The cultivation of the d-xylitol-producing *A. niger* strain was carried out with a synthetic medium containing 12 g L^−1^ d-glucose as a carbon source and 13 g L^−1^ l-arabinose as the educt. The stirred tank bioreactors were inoculated with 10^9^ spores L^−1^. The CO_2_ and O_2_-concentrations were measured in the exhaust gas to monitor the metabolic activity of the engineered *A. niger* strain. Maximum metabolic activities were measured after 40 h, and 42 h, respectively (Figure 1). Pellet formation of filamentous fungi is often heterogeneous and it could be a cause for the slight time shift between both processes [19]. Aeration of the stirred tank bioreactors was stopped automatically after complete l-arabinose consumption, indicated by the rapid decrease in carbon dioxide concentration in the exhaust gas after 70 h, and 76 h, respectively. Directly after finishing aeration, the stirred tank bioreactors were flushed automatically with nitrogen gas (N_2_) for two minutes to inactivate the metabolism of the fungi and to reduce the oxidative potential of the cells, which prevents the conversion of d-xylitol to d-xylulose. This control strategy was implemented to prevent re-consumption of d-xylitol by the engineered *A. niger* strain after the depletion of l-arabinose in the batch processes (Figure S1) [17].

**Figure 2:**
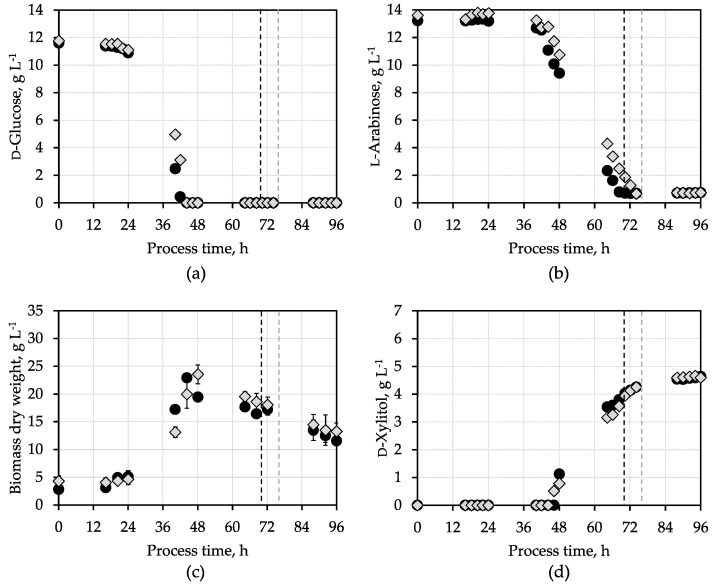
d-Xylitol production by engineered *A. niger* in stirred tank bioreactors with synthetic medium: Concentrations of (**a**) d-glucose, (**b**) l-arabinose, (**c**) biomass dry weight, and (**d**) d-xylitol in two parallel batch processes with the d-xylitol-producing *A. niger* strain (pH 4.5, 30 °C, 0.2 vvm aeration after 3 h until the total consumption of l-arabinose was detected online by the rapid decrease in CO_2_ production, indicated by the dashed lines).

The sugar and biomass dry weight (BDW) concentrations of these batch processes are shown in Figure 2. The energy and carbon source d-glucose was depleted within 44 h in both processes and matched the drop in the exhaust gas at that time. The consumption of l-arabinose started after d-glucose depletion. l-arabinose was nearly completely consumed after 70 h, and 76 h, which matches the CO_2_ concentration drop in exhaust gas data (Figure 1). In both batch processes, about 0.7 g L^−1^ l-arabinose remained in the medium, and accordingly, about 12 g L^−1^ l-arabinose was consumed after the aeration was stopped automatically. d-xylitol formation started parallel to l-arabinose consumption. However, after the automated switch-off of the aeration, further d-xylitol accumulation was observed, although at reduced rates without consumption of the remaining l-arabinose (Figure 2). This phenomenon might, therefore, be caused by delayed export of intracellular d-xylitol via the Fps1 transporter. Kinetic data on d-xylitol transport by the Fps1 transporter, usually used for glycerol transport to regulate osmotic stress tolerance in *S. cerevisiae* [17], are missing so far.

The maximum biomass concentrations of 22.9 ± 0.8 g L^−1^ and 23.5 ± 1.7 g L^−1^, respectively, were measured shortly after the maximum metabolic activities occurred in both batch processes with biomass yields of 0.58 ± 0.07 g BDW g^−1^ glucose and 0.61 ± 0.14 BDW g^−1^ glucose. Afterward, a linear decay of the BDW concentrations was observed during d-xylitol production, indicating constant lysis of the engineered *A. niger* cells. Finally, after 96 h, the biomass concentrations were considerably reduced to 11.6 ± 0.9 g L^−1^ and 13.2 ± 1.5 g L^−1^ BDW, respectively.

The estimated final d-xylitol yields of 0.36 g g^−1^ and 0.37 g d-xylitol g^−1^ l-arabinose are smaller compared to the yield of 0.45 g d-xylitol g^−1^ l-arabinose measured in shake flasks with the same engineered *A. niger* strain [17]. The re-consumption of d-xylitol seems less pronounced on a shake flask scale compared to the stirred tank bioreactor cultivations at controlled conditions.

### 3.2. d-Xylitol Production Using SBPP Hydrolysate

Batch cultivations of the d-xylitol-producing *A. niger* strain were carried out with SBPP hydrolysate produced in a one-pot process with *A. niger* containing 13 g L^−1^ l-arabinose, 9 g L^−1^ d-glucose, 9 g L^−1^ d-galacturonic acid, and 4.5 g L^−1^ xylose, mannose, and galactose [9].

In the first approach, the SBPP hydrolysate was filtered and heat sterilized at 120 °C for 20 min before use, and the stirred tank bioreactors were inoculated with 10^9^ spores L^−1^ of the d-xylitol-producing *A. niger* strain. Surprisingly, there was no sugar consumption and no growth of the engineered *A. niger* within 168 h.

In a second approach, the batch cultivation of the d-xylitol-producing *A. niger* strain was conducted using SBPP hydrolysate without heat sterilization. In addition, the stirred tank bioreactor was inoculated with active fungi from a shake flask preculture to avoid any negative effects of the SBPP hydrolysate on germination of the *A. niger* spores. However, also in this case, no growth or sugar consumption occurred. This clearly indicated that the SBPP hydrolysate produced by *A. niger* in a one-pot process contains inhibiting substances after centrifugation, filtration, and storage at −20 °C, which prevented the growth of the d-xylitol-producing *A. niger*.

The production of growth-inhibiting by-products from lignin-degradation may occur during hydrolysis [20,21,22]. SBPP’s lignin content is reported as 4.41% or 5.5% [23,24]. The inhibitory by-products released from SBPP through acid hydrolysis, not applied in this study, include furan derivatives (such as furfural and 5-hydroxymethyl furfural), phenolic compounds, and weak acids. In a study, the hydrolysate after acid hydrolysis of SBPP contained 45.3 g L^−1^ total reducing sugars, 514.5 mg L^−1^ total furan compounds, 2.9 g L^−1^ phenolic compounds, and 3.8 g L^−1^ acetic acid [25]. Concentrations known to inhibit 50% of an *Aspergillus* subspecies growth are 1.68 g L^−1^ (or 17.12 mM) furfural and 0.19 g L^−1^ (or 1.51 mM) hydroxymethyl furfural [26]. The predominant phenolic compounds in SBPP are epicatechin, gallic acid, and quercetin-3-O-rutinoside. Phenolic compounds with antifungal activities in sugar beet extract include ferulic acid and hydroxycinnamic acid-type phenols [27]. Another publication determined the antifungal activity of different phenolic compounds. Gallic acid inhibits at concentrations above 1 mg L^−1^, while ferulic acid inhibits 50% of the growth at a concentration of 0.895 mg L^−1^ [28].

Enzymatic hydrolysis of SBPP was performed at 50 °C in the one-pot process [9]. Several studies have found *Ascomycete* fungi, including *Aspergilli*, to be able to (partially) degrade lignin, which could have contributed to the release of some potentially inhibiting lignin-derived compounds during the hydrolysis at 50 °C [29,30].

Detoxification of SBPP hydrolysate has been shown in various studies with activated charcoal after thermo-chemical hydrolysis before its use in fermentation [25,31]. According to the literature, pre-treatment with 20 g L^−1^ activated charcoal removed 98% of furan compounds, 71.1% of phenolic compounds, and 10% of acetic acid [25]. In another study, the treatment with 5 g L^−1^ activated charcoal removed 98% of color from beet molasses without co-adsorption of sucrose and betaine [32]. However, a further study showed a 10.2% total sugar loss with 5% activated charcoal loadings to remove inhibiting by-products from lignocellulosic hydrolysates [33]. Sugar loss was observed as well after detoxification of agave tequilana bagasse hydrolysate with 10% (*w/v*) activated charcoal resulting in a sugar recovery rate of only 74.4% [34].

### 3.3. d-Xylitol Production Using Activated Charcoal Pre-Treated SBPP Hydrolysate

**Figure 3:**
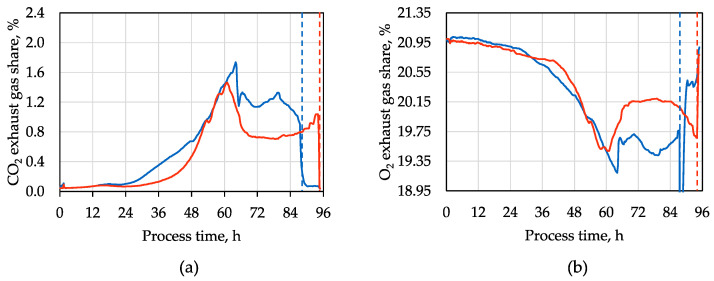
d-Xylitol production by engineered *A. niger* in stirred tank bioreactors with pre-treated SBPP hydrolysate: (**a**) carbon dioxide (CO_2_), and (**b**) oxygen (O_2_) concentrations in the exhaust gases of two parallel batch processes with the d-xylitol-producing *A. niger* strain (pH 4.5, 30 °C, 0.2 vvm aeration after 3 h until the total consumption of l-arabinose was detected online by the rapid decrease in CO_2_ production, after 86 h and 95 h, respectively, with subsequent aeration switch-off indicated by the dashed lines; red line: spore loss during inoculation).

For the next batch cultivations of the d-xylitol-producing *A. niger* strain with clarified SBPP hydrolysate, the hydrolysate was pre-treated with 75 g activated charcoal L^−1^ SBPP hydrolysate to test whether the inhibiting substances can be removed. This pre-treatment first removed the dark color of the SBPP hydrolysate (Figure S2). The sugar recovery after the pre-treatment was 95.5% d-glucose, 95.7% l-arabinose, 92.8% d-galacturonic acid, and 98.3% xylose, mannose, and galactose. The pre-treated SBPP hydrolysate contained 11 g L^−1^ d-glucose, 13 g L^−1^ l-arabinose, 12 g L^−1^ d-galacturonic acid, and 5 g L^−1^ xylose, mannose, and galactose. The stirred tank bioreactors were inoculated with 10^9^ spores L^−1^ of the d-xylitol-producing *A. niger* each and gassed with sterile air. In one of the replica processes (red line in Figure 3), some spore loss occurred during inoculation due to a leaking syringe. The CO_2_ and O_2_-concentrations were measured in the exhaust gas to monitor the metabolic activity of the *A. niger* strain (Figure 3). The fungi were growing with the pre-treated SBPP hydrolysate, and maximum metabolic activities were observed after 60 h (red), and 64 h (blue), respectively. The automated aeration stopped after the drop in the CO_2_ concentration in the off-gas (dashed lines in Figure 3).

The process performance data are compared to the synthetic medium’s batch cultivations shown before (Figure 2). The pre-treated SBPP hydrolysate resulted in a delay of the maximal metabolic activities of the d-xylitol-producing *A. niger* cells of about 24 h compared to the synthetic medium. The exhaust gas concentration dynamics differ between the synthetic and hydrolysate medium due to the additional sugars in the SBPP hydrolysate. A distinct difference is also observed between the two batch processes using hydrolysate, which can be attributed to variations in pellet morphology (Figure S3). This morphological variation is, in turn, most likely linked to the spore loss that occurred during inoculation of one of the batch processes, because the spore density at inoculation impacts morphology [19]. However, the maximum CO_2_ concentrations in the off-gas were comparable.

**Figure 4:**
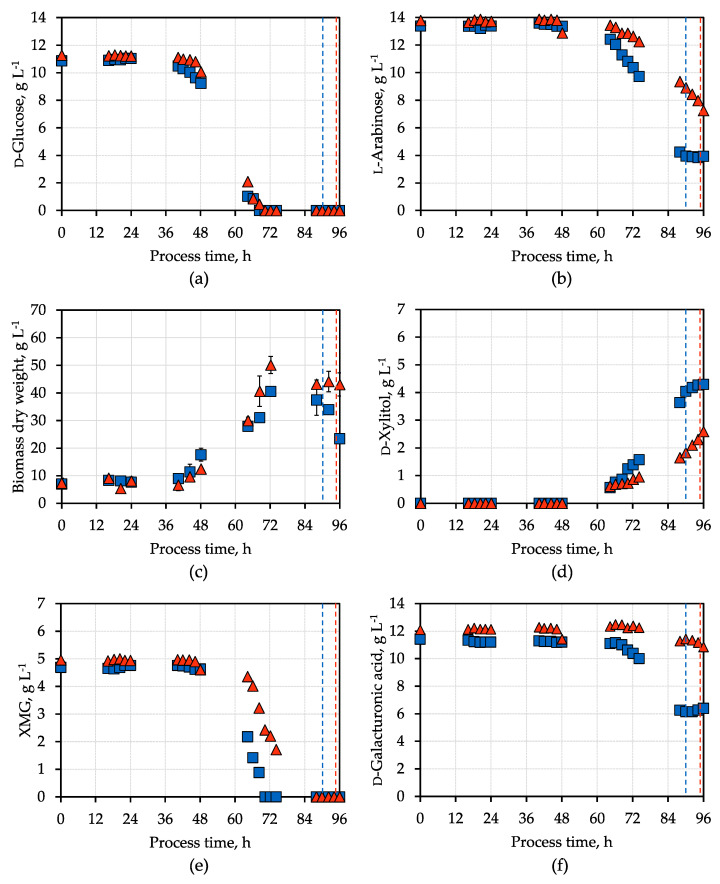
d-Xylitol production by engineered *A. niger* in stirred tank bioreactors with SBPP hydrolysate: concentrations of (**a**) d-glucose, (**b**) l-arabinose, (**c**) biomass dry weight, (**d**) d-xylitol, (**e**) xylose, mannose, galactose (XMG), andd(**f**) d-galacturonic acid in two parallel batch processes with the d-xylitol-producing *A. niger* strain (pH 4.5, 30 °C, 0.2 vvm aeration after 3 h until the rapid decrease in CO_2_ production rate, indicated by the dashed lines; blue symbols: no spore loss; red symbols: spore loss during inoculation).

The sugar and the BDW concentrations in the batch processes with pre-treated SBPP hydrolysate are shown in Figure 4. d-Glucose was depleted within 87 h (blue) and 95 h (red) and matched the rapid drop of CO_2_ in the exhaust gas at that time. The consumption of l-arabinose started after d-glucose depletion. As before, the d-xylitol formation started with the consumption of l-arabinose. Surprisingly, l-arabinose was not fully consumed when the CO_2_ evolution rate rapidly decreased with pre-treated SBPP hydrolysate (Figure 4, blue symbols). After the aeration was switched off automatically, the l-arabinose and d-xylitol concentrations remained constant. The final d-xylitol concentrations were 4.3 g L^−1^ and 2.6 g L^−1^ (spore loss during inoculation) after a process time of 96 h. The final d-xylitol yields were 0.45 g g^−1^ and 0.4 g g^−1^ (spore loss during inoculation) l-arabinose, respectively.

The maximum BDW concentrations of 40.5 ± 1.6 g L^−1^ and 50.1 ± 3.1 g L^−1^ were observed after 72 h in both batch processes after glucose was depleted. Biomass formation is significantly enhanced when using SBPP hydrolysate compared to the synthetic medium. The consumption of glucose predominantly occurs in parallel with the utilization of other sugars xylose, mannose, and galactose (XMG), leading to increased biomass production. The d-galacturonic acid is only metabolized once all other sugars, except l-arabinose, have been depleted. This conversion remains incomplete and halts when aeration is stopped.

The key process performance indicators of the cultivation experiments using synthetic medium and pre-treated SBPP hydrolysate are compared in Table 1. The maximum mean CO_2_ concentration was higher and 20 h earlier with the synthetic medium than SBPP hydrolysate, indicating that there might have been some inhibiting components left in the SBPP after activated charcoal treatment, causing the extended lag phase. On the other hand, the maximum BDW concentration was about 22.1 g L^−1^ higher with the pre-treated SBPP hydrolysate. The increased BDW is likely due to additional sugars in the hydrolysate, enabling increased biomass formation and d-galacturonic acid consumption parallel to d-xylitol formation. SBPP hydrolysate contains, in addition to a variety of fermentable sugars, other nutrients that can support robust microbial growth [35,36]. Comparing the final d-xylitol yields, they were 0.06 g g^−1^ higher with the SBPP hydrolysate medium compared to the synthetic medium. However, the final d-xylitol concentration was lower with hydrolysate since l-arabinose was not fully consumed. Still, the d-xylitol yields achieved with the engineered *A. niger* with l-arabinose were considerably lower compared to the yield of 0.9 mol d-xylitol mol^−1^ d-xylose reported with an engineered *S. cerevisiae* strain using a synthetic medium with d-xylose as the substrate [37].

The increased BDW concentration did not lead to an increased l-arabinose conversion rate, while the d-xylitol accumulation is probably linked to the FPS1 transporter export rate. However, the yield is higher with SBPP hydrolysate, most likely due to the presence of d-galacturonic acid as an additional carbon source, since the fungi can still consume it in parallel to the d-xylitol formation. This is also indicated by the constant BDW concentration after glucose and XMG depletion in both SBPP hydrolysate processes. Conversely, after the aeration stop with SBPP hydrolysate, the BDW concentration decreases, despite the remaining sugars in the medium, because of the oxygen limitation.

While the aeration stop also worked well to prevent d-xylitol depletion with the SBPP hydrolysates, it occurred too early in both batch processes. The CO_2_ concentration threshold of 0.9% CO_2_ in the exhaust gas, which should indicate l-arabinose depletion coming soon, was too high for the automated stop of the aeration. This threshold was chosen based on the assumption that more CO_2_ will be produced by the engineered *A. niger* cells due to the parallel consumption of d-galacturonic acid in the SBPP hydrolysate. Consequently, it is crucial to identify a suitable CO_2_ threshold in the exhaust gas in further studies for the automated aeration switch-off with SBPP hydrolysate. An alternative will be to implement a time-delayed switch-off.

## 4. Conclusions

The production of d-xylitol from clarified SBPP hydrolysate using the engineered *A. niger* strain successfully demonstrates the feasibility of utilizing agricultural residue SBPP as a substrate for d-xylitol production. This approach provides a foundation for future research on SBPP valorization, although further studies are required to fully establish its potential.

Surprisingly, a pre-treatment of the enzymatically produced SBPP hydrolysate was necessary, most probably due to the enzymatic hydrolysis at 50 °C in the one-pot enzyme production and hydrolysis upstream process with *A. niger* [9]. The pre-treatment with activated charcoal proved to be a practical approach for detoxifying the SBPP hydrolysate with minimal sugar loss, making it useful for further conversion with *A. niger*. The d-xylitol yield of 0.43 ± 0.03 g d-xylitol g^−1^ l-arabinose using the pre-treated SBPP hydrolysate was significantly higher compared to batch processes using synthetic medium (0.36 ± 0.01 g g^−1^), most likely due to the availability of other sugars in the hydrolysate.

The aeration switch-off was shown to be an easily implementable and successful online control to prevent further d-xylitol consumption after l-arabinose depletion, but it needs adaption to the sugar composition of the hydrolysate. A promising approach to improve the automatic aeration stop could consider both the CO_2_ concentration and the detection of a gradient decline in CO_2_ evolution.

First, further research is needed to identify the SBPP hydrolysate’s inhibitory compounds. Second, dynamic process analysis is necessary to measure when they are formed during the upstream one-pot process for enzyme production and hydrolysis. Third, a search for modifications of the upstream one-pot process should be carried out to reduce the formation of the most inhibitory compounds.

Due to the low final product concentration, d-xylitol isolation will be the next challenging task. This is because the l-arabinose yield of SBPP hydrolysis is solely 0.18 g g^−1^, resulting in a total d-xylitol yield of 0.08g g^−1^ SBPP after hydrolysis, pre-treatment, and d-xylitol production. Recent studies have suggested d-xylitol purification by combining ion exchange chromatography followed by crystallization [38,39].

## Figures and Tables

**Table 1:** Comparison of the key process performance indicators of the d-xylitol production processes performed with synthetic medium and with pre-treated SBPP hydrolysate medium with engineered *A. niger.* The indicators of the process with the synthetic medium are the mean values and with SBPP medium not (process values with spore loss during inoculation are added in brackets).

Parameter	Synthetic Medium	SBPP Hydrolysate Medium
Maximal CO_2_ value, %	1.67 ± 0.13	1.71 (1.46)
Maximal CO_2_ value time, h	42 ± 1	64 (61)
Maximal BDW concentration, g L^−1^	23.2 ± 0.3	40.5 ± 1.6 (50.1 ± 3.1)
Maximal BDW concentration time, h	46 ± 2	72 (72)
Final d-xylitol concentration, g L^−1^	4.62 ± 0.02	4.3 (2.6)
Final d-xylitol yield, g g^−1^	0.36 ± 0.01	0.45 (0.4)

## Data Availability

The original contributions presented in the study are included in the article; further inquiries can be directed to the corresponding authors.

## Supplementary Materials

The following supporting information can be downloaded at: https://www.mdpi.com/article/10.3390/microorganisms12122489/s1, Figure S1: d-xylitol production by engineered *A. niger* in stirred tank bioreactors with synthetic medium: Concentrations of (a) CO_2_ in the exhaust gas, (b) d-glucose, (b) l-arabinose, (c) biomass dry weight, and (d) d-xylitol in two parallel batch processes with the d-xylitol-producing *A. niger* strain (pH 4.5, 30 °C, initial 0.2 vvm aeration and gradually increased to keep the dissolved oxygen concentration above 30%); Figure S2: Color change of SBPP hydrolysate before (a) and after activated charcoal treatment (b); Figure S3: Morphology of engineered *A. niger* strain with SBPP hydrolysate medium (a,c) and synthetic medium (b,d) after 68 h process time.
