# Supplementary material for: d-Xylitol Production from Sugar Beet Press Pulp Hydrolysate with Engineered Aspergillus niger
Source: Microorganisms. 2024 Dec 3;12(12):2489. doi: 10.3390/microorganisms12122489 (PMC11727921; doi:10.3390/microorganisms12122489)
Supplement: Supplementary file 1 [file microorganisms-12-02489-s001.zip › microorganisms-3337505-supplementary.pdf]

Supplementary material.

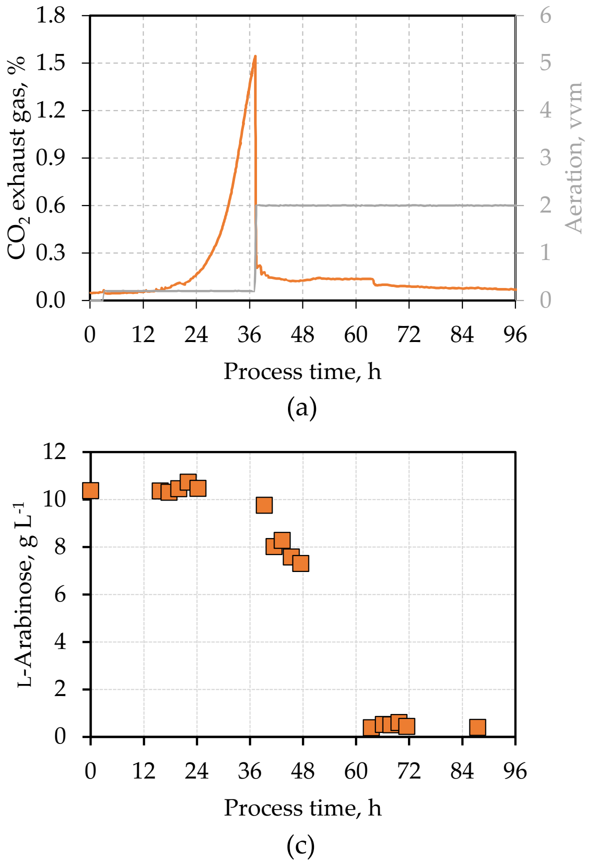

Figure S1. D-Xylitol production by engineered *A. niger* in stirred tank bioreactors with synthetic medium: Concentrations of (**a**) CO_2 in the exhaust gas, (**b**) D-glucose, (**b**) L-arabinose, (**c**) biomass dry weight, and (**d**) D-xylitol in two parallel batch processes with the xylitol-producing *A. niger* strain (pH 4.5, 30 °C, initial 0.2 vvm aeration and gradually increased to keep the dissolved oxygen concentration above 30%).

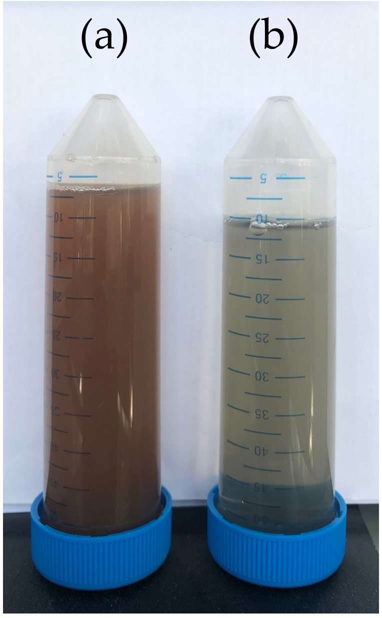

Figure S2. Color change of SBPP hydrolysate before (**a**) and after activated charcoal treatment (**b**).

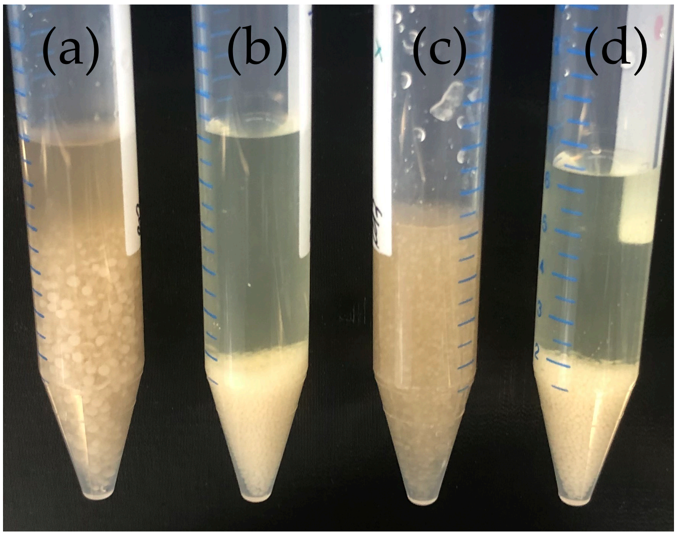

Figure S3. Morphology of engineered *A. niger* strain with SBPP hydrolysate medium (**a**,**c**) and synthetic medium (**b**,**d**) after 68 h process time.
